# Development of a New Affinity Gold Polymer Membrane with Immobilized Protein A

**DOI:** 10.3390/membranes14020031

**Published:** 2024-01-24

**Authors:** Tobias Steegmüller, Tim Kratky, Lena Gollwitzer, Sebastian Patrick Schwaminger, Sonja Berensmeier

**Affiliations:** 1 Chair of Bioseparation Engineering, TUM School of Engineering and Design, Technical University of Munich, Boltzmannstraße 15, 85748 Garching, Germany; tobias.steegmueller@tum.de (T.S.);; 2 Associate Professorship Physical Chemistry with Focus on Catalysis, TUM School of Natural Sciences, Technical University of Munich, Lichtenbergstraße 4, 85748 Garching, Germany; tim.kratky@tum.de; 3 Division of Medicinal Chemistry, Otto-Loewi Research Center, Medical University of Graz, Neue Stiftingtalstraße 6, 8010 Graz, Austria; 4 BioTechMed-Graz, Mozartgasse 12, 8010 Graz, Austria; 5 Munich Institute of Integrated Materials, Energy and Process Engineering, Technical University of Munich, Lichtenbergstraße 4a, 85748 Garching, Germany

**Keywords:** affinity membrane chromatography, protein A, antibody purification, protein immobilization, gold membrane

## Abstract

New and highly selective stationary phases for affinity membrane chromatography have the potential to significantly enhance the efficiency and specificity of therapeutic protein purification by reduced mass transfer limitations. This work developed and compared different immobilization strategies for recombinant Protein A ligands to a gold-sputtered polymer membrane for antibody separation in terms of functionalization and immobilization success, protein load, and stability. Successful, functionalization was validated via X-ray photoelectron spectroscopy (XPS). Here, a recombinant Protein A ligand was coupled by N-hydroxysuccinimide (NHS)/N-(3-dimethylaminopropyl)-N′-ethylcarbodiimide (EDC) chemistry to carboxy-functionalized, gold-sputtered membranes. We achieved a binding capacity of up to 104 ± 17 mg of the protein ligand per gram of the gold-sputtered membrane. The developed membranes were able to successfully capture and release the monoclonal antibody (mAb) Trastuzumab, as well as antibodies from fresh frozen human blood plasma in both static and dynamic setups. Therefore, they demonstrated successful functionalization and immobilization strategies. The antibody load was tested using bicinchoninic acid (BCA), ultraviolet-visible spectroscopy (UV-vis) measurements, and sodium dodecyl sulfate polyacrylamide gel electrophoresis (SDS-PAGE). The outcome is a fully functional affinity membrane that can be implemented in a variety of different antibody purification processes, eliminating the need for creating individualized strategies for modifying the surface to suit different substrates or conditions.

## 1. Introduction

Membrane chromatography is a promising method for bioseparation engineering. It combines the procedures of membrane filtration and chromatography [1,2,3]. The method works on the idea of a molecule adsorbing to the stationary phase, the membrane, and others flowing through without harming the adsorbed molecule. The adsorbed entity is then eluted via ionization, pH change, and other methods. Membrane chromatography offers various advantages as a protein separation technology. Because of the convective flow, mass transfer resistance is considerably decreased, enabling binding kinetics to be the primary driver of the adsorptive process. This leads to flow-independent dynamic binding capacities and allows faster processing, which lowers the residence time and biomolecule inactivation. Higher flow rates, productivity, simplicity of packing, and scale-up are only a few of the many benefits membrane adsorbers have over conventional packed-bed chromatography. Recent studies explored the utility of membranes as stationary phases in chromatographic system for high-throughput protein purifications as well as analytical separations of biomolecules [1,2,4]. The overall binding capacity, however, is restricted due to the membrane’s small specific surface area [5]. For antibody purification, only one commercial product, the Sartobind^®^ affinity membrane from Sartorius (Göttingen, Germany) is available, with a dynamic binding capacity (i.e., DBC10) of 5–7.5 mg mL^−1^ [6,7].

Although membrane chromatography provides several advantages, in order to facilitate specific target protein adsorption to the stationary phase (i.e., an affinity chromatographic process), the challenge is to change the material without compromising its endurance [8]. Different polymeric materials can be used in functionalization approaches, such as polyamide (PA), polypropylene (PP) [9], polyethersulfone (PES) [10], and cellulose [11]. However, the harsh chemical conditions of common antibody purification procedures also establish certain parameters (i.e., acid and base resistance) for the construction of a membrane chromatography procedure. The material must be able to be immobilized with a proteinogenic ligand and be resistant to harsh pH changes. In this study, an eightfold polymerized B domain from the native Protein A, which is C-terminally fused with the octapeptide (HR)_4_-tag for purification reasons is used [12,13]. As a consequence of its eight-fold homo-polymerization properties, the ligand demonstrates an enhanced binding stoichiometry to IgG, thereby yielding higher binding capacities [14]. Covalent immobilization of proteins has been shown to increase its pH stability [15]. Polyethersulfone (PES) is a well-known and established membrane material. It is beneficial due to its chemical-, mechanical resistance, and thermal stability [16,17]. The material used in this study is a gold-sputtered PES membrane. The gold layer offers additional chemical stability to the polymer material even at a low pH, as well as low toxicity, making it a suitable candidate for therapeutic protein purification processes [18,19], and it reduces membrane fouling [20]. PES alone, however, has some disadvantages due to its hydrophobicity, which is one of the leading causes of membrane biofouling. A chemical modification of the material can increase its hydrophilicity or introduce new functional groups in order to obtain the desired effects [21]. A common modification method for polyethersulfone is the grafting of carboxylic groups [17]. The introduction of carboxylic groups increased the hydrophilicity of the material, enabling a functional matrix suitable for an N-hydroxysuccinimide/N-(3-dimethylaminopropyl)-N′-ethylcarbodiimide (NHS/EDC)-coupling reaction and consecutively for protein immobilization [22]. This functionalization strategy is in principle applicable to all gold-sputtered materials, whereas the functionalization of the native membrane materials would require an adaptation to each different polymer used [9]. However, the functionalization of the whole membrane material would be less prone to degradation and could provide a higher surface area and more chemically stable functionalization [9,17], which is especially important during the harsh elution process conditions (i.e., Na-acetate at a pH of 2.9 as the elution buffer [23]).

**Figure 1:**
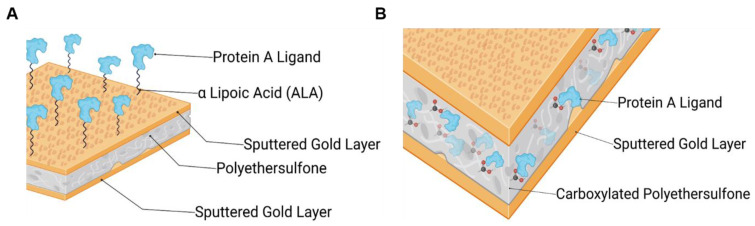
Schematic of immobilized Protein A ligand via two different functionalization approaches, created via Biorender: (**A**) via a self-assembled monolayer of ALA on the gold surface (ALA@Au_PES200) and (**B**) via carboxy groups grafted into PES by e beam technology (Au_PES200@COOH).

This study aimed to develop and evaluate two different approaches to immobilize a Protein A ligand onto gold-sputtered PES membranes and to compare their performance to a commercially available Protein A membrane, Sartobind^®^ by Sartorius (Göttingen, Germany). One aimed for the introduction of carboxylic groups via a self-assembled monolayer on the gold-sputtered surface (see Figure 1A), and the other one involved a PES material that was functionalized via an electron beam technology by qCoat (see Figure 1B). The goal was to modify the inert gold-sputtered PES membrane by adding carboxylic groups to enable NHS/EDC protein immobilization, followed by immobilizing a Protein A ligand to the membrane. The resulting membranes were tested in both static and dynamic experiments to assess their applicability in a process, and their functionality was demonstrated using a complex medium such as human blood plasma. With this work, a functional affinity membrane could be created out of an inert gold-sputtered PES membrane material, that could be implemented in a variety of new assays and technologies—such as potential-controlled affinity chromatography or potential-controlled drug release.

## 2. Materials and Methods

### 2.1. Materials

All solvents and chemicals used were of analytical grade. Buffers for analytical and preparative purposes were filtered (0.2 µm diameter) and degassed. The tenfold phosphate-buffered saline stock buffer was purchased from Rockland, NY, USA, and consisted of 0.2 M KPO_4_, 1.5 M NaCl, and 0.1% (*w*/*v*) Na-azide. A tenfold dilution of the 10× PBS stock buffer with water resulted in a 1× PBS buffer at pH 7.4. All reagents were purchased from Sigma Aldrich (Taufkirchen, Germany); for SDS-PAGE, all reagents were from Thermofisher (München, Germany). The fluorescence protein dye Atto 488 was purchased from Jena Bioscience, and the PD-10 gel filtration column with Sephadex G-25 was purchased from Cytiva (Uppsala, Sweden). The cloning and expression of the B8(RH)_4_ ligand was published earlier by our group [23]. The ligand protein B8 was purified via the affinity tag and NiNTA chromatography and polished via size exclusion chromatography; commercial recombinant Protein A was purchased from Sino Biological (Eschborn, Germany). The monoclonal antibody Trastuzumab was supplied as cell culture supernatant from Roche (Penzberg, Germany) and was purified with a Bio-Scale™ Mini UNOspere SUPrA™ 5 mL Protein A column from Bio-Rad (Feldkirchen, Germany). All protein purifications were performed on an ÄKTApure system from Cytiva (Uppsala, Sweden) and purities were assessed via SDS-PAGE densitometric analysis, prior to their use in any experiment. Fresh frozen human blood plasma was supplied by the Bavarian blood donation service of the Red Cross. Membranes were provided by i3 Membrane GmbH (Hamburg, Germany); the gold-sputtered polyethersulfone (PES) membrane with an average pore diameter of 200 nm is abbreviated as Au_PES200, and the carboxylated PES membranes from i3 Membrane and qCoat are abbreviated as Au_PES200@COOH (Figure 1B). Au_PES200 membranes functionalized via α-lipoic acid (ALA) (Figure 1A) are abbreviated as ALA@Au_PES200. The gold-sputtered membranes offer a 100 nm thick gold coating on each side and, according to the supplier i3 Membrane GmbH, a 100 µm layer of PES. The industry standard membrane used for comparison was isolated from a stacked module of Sartobind^®^ Protein A membranes (Göttingen, Germany). The membrane material used here was a regenerated cellulose material with an average pore diameter of 450 nm, which was functionalized with recombinant Protein A. For all dynamic experiments, a 25 mm Whatman^®^ plastic filter holder module from Merck (Darmstadt, Germany) was used, capable of holding membranes with a diameter of 22 mm. For fluorescence detection and SDS-PAGE analysis, an Amersham Typhoon scanner from Cytiva (Uppsala, Sweden) was used, as well as for the quantification of Imagequant software (version, v8.2.0.0). For BCA assays and UV-vis, a multiplate reader from the Tecan Group (Männedorf, Switzerland) and a NP-80 photometer from Implen (München, Germany) were used, respectively. For the dynamic experiment via FPLC, the filter holder module was connected to an ÄKTApure system (Uppsala, Sweden).

### 2.2. Experimental

#### 2.2.1. Functionalization of the Membranes

The functionalization processes are important to make the inert PES membranes accessible for later protein immobilization. For this, two different approaches can be undertaken: the functionalization of the gold surface via α-lipoic acid (ALA) or the direct functionalization of the membrane material via e beam (see Figure 1).

1. Functionalization of the gold surface (see Figure 1A) via alphalipoic acid (ALA): Alphalipoic acid undergoes a process of self-assembled monolayer formation [24] due to a thiol-gold bond. Therefore, any gold surface can be carboxylated. For the functionalization, a 2% (*w*/*v*) solution of alphalipoic acid in ethanol was prepared. The respective membrane pieces were then incubated at 22 °C for 24 h at 500 rpm in a tabletop shaker. After incubation, the membranes were washed three times with ethanol and dried with compressed air.
2. Functionalization of carboxylated PES membranes (see Figure 1B): The gold-sputtered PES membranes were carboxylated via an electron beam (e beam) process.

#### 2.2.2. Analysis of Functionalization via XPS

X-ray photoelectron spectra were recorded on a Leybold-Heraeus LHS 10 spectrometer using a non-monochromatized Mg Kα source (1253.6 eV). The membrane samples were mounted on a sample holder, and the analyzed front was grounded. All spectra were recorded in an ultra-high vacuum chamber at a pressure below 2 × 10^−8^ mbar. The analyzer was operated at a constant pass energy of 100 eV, leading to an energy resolution with a full width at half-maximum (fwhm) of ~1.1 eV. While the gold-sputtered PES200 membranes did not show any sample charging, the energy scales of the native PES200 spectra were corrected by using the C 1’s main signal at 285.0 eV [25].

#### 2.2.3. Protein Immobilization via NHS-EDC

**Figure 2:**
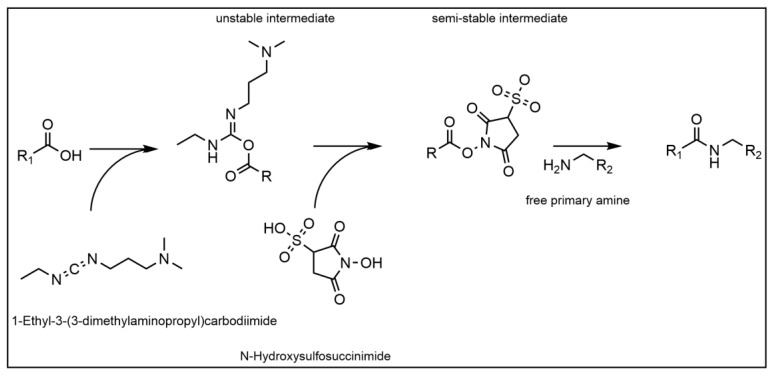
Reaction mechanism of the NHS/EDC reaction.

In order to immobilize the recombinant Protein A ligand covalently to the membranes, the NHS/EDC reaction was chosen (see Figure 2).

The reaction offers a stable covalent bond (i.e., amid bond) formation between a proteins N-terminus and a carboxyl-group on the stationary phase. However, two different NHS/EDC reactions were screened as well, in order to establish the most suitable process conditions for a highly selective candidate. For the Au_PES200@COOH membranes, two different protein immobilization protocols were used and tested against each other. For the ALA functionalized ALA@Au_PES200 membranes, only immobilization protocol (I) was used, as follows:

(I) For the immobilization, the membranes were washed four times with an activation buffer (50 mM of 2-(N-morpholino)ethanesulfonic acid (MES) buffer at pH 6.0), following an immobilization protocol outlined by Merck Millipore [26]. After washing, 24 µL of an aqueous 200 mM 1-ethyl-3-(3-dimethylaminopropyl) carbodiimide solution (EDC) was supplemented per mL of activation buffer. Immediately after the EDC-solution, 240 µL per mL of activation buffer of a 200 mM N-hydroxysulfosuccinimide (sulfo-NHS) solution (dissolved in activation buffer) was added. The mixture was incubated for 30 min at room temperature at 600 rpm. After incubation, the supernatant was removed, and the membranes washed three times with the activation buffer. The activated membranes were then immersed in a 1 mg mL^−1^ Protein A in the activation buffer solution and incubated at 25 °C for 2.5 h at 600 rpm. After incubation, 50 µL ethanolamine were added and incubated for 30 min to stop the reaction. The supernatant was removed, and the membranes were immersed in a 50 mM Tris(hydroxymethyl)aminomethane (Tris) 0.5% (*w*/*v*) casein blocking buffer at pH 8.0 and incubated at room temperature overnight at 600 rpm. The supernatant was removed, and the membranes washed twice with blocking buffer.
(II) During a second immobilization approach following a protocol from G-Bioscience [27], the membranes were immersed in a 0.1 M MES buffer at pH 4.6 and supplemented with an aqueous solution of 0.4 mg mL^−1^ EDC and 0.6 mg sulfo-NHS. The mixture was incubated for 15 min at 600 rpm at room temperature. To stop the reaction, 1.5 µL of 3-mercaptoethanol was added before immersing the membranes in a 1 mg mL^−1^ Protein A in 50 mM NaH_2_PO_4_, 150 mM NaCl solution at pH 7.4. The immersed membranes were incubated for 2 h in the solution before quenching the reaction with 0.5 M hydroxylamine.

Both immobilization strategies were tested in a static and dynamic binding setup, and the binding were capacities compared against each other.

#### 2.2.4. Fluorescence Labeling

To visualize the successful immobilization of Protein A, the target antibody Trastuzumab was N-terminally labeled with an Atto 488 NHS-ester and scanned via a fluorescence scan. The labeled antibody (*) binds to the ligand protein and can be quantified via a fluorescence intensity comparison via the Imagequant software (version, v8.2.0.0). The antibodies were labeled following the protocol by Jena Bioscience [28]. In total, 1 mL deionized water was combined with 1 M NaHCO_3_. The monoclonal antibody (mAb) concentration was adjusted to 100 mM using this solution. Next, 100 µL of N,N-dimethylformamide (DMF) was vortexed with the 10 mg mL^−1^ dye. Then, 100 µL of the protein solution (10 mg mL^−1^) and 10 µL dye (10 mg mL^−1^) were combined and centrifuged. This was followed by a 1 h incubation period in a light-protected shaker at room temperature. The PD-10 desalting column was used to remove unreacted dye from the protein solution. The resulting conjugate was analyzed via UV-vis spectroscopy via a NP-80 photometer, and the degree of labeling (DOL) was determined via Equation (1).

(1) $$DOL=\frac{A_{max}\times \varepsilon_{280}}{\left(A_{280}-A_{max}\times CF\right)\times \varepsilon_{max}}$$

where A_max_ represents the absorbance of the fluorescently labeled Trastuzumab at a wavelength of 501 nm, A_280_ the absorbance of the labeled protein at 280 nm, CF the correction factor of the Atto 488 dye of 0.1, ε_280_ the extinction coefficient for Trastuzumab of 210,000 cm^−1^ M^−1^, and ε_max_ the extinction coefficient of the Atto 488 dye of 90,000 cm^−1^ M^−1^. The labeled antibody was supplied to the previously functionalized membranes at a concentration of 0.5 mg mL^−1^ and incubated for 1 h at room temperature. The membranes were then washed three times with a 1× PBS buffer at pH 7.4 before being placed into the scanner. A CY2 emission filter was used for the fluorescence imaging measurements, as the 532 nm laser is used to excite the Atto 488 dye, to separate laser light from fluorescence [29].

#### 2.2.5. Static Experiments

Each treated membrane was placed in a defined Trastuzumab solution of varying concentrations to estimate the q_max_, via a Langmuir isotherm fitting. Trastuzumab concentrations of 0.5, 0.3, 0.2, 0.1, 0.05, 0.025, 0.01, and 0 mg mL^−1^ in PBS pH 7.4 were prepared. The membranes were cut to a 6 mm diameter, placed in 0.25 mL of each concentration, and incubated for 1 h at 25 °C and 1000 rpm. After this, a supernatant analysis and direct analysis of the membranes occurred via UV-vis and BCA assay, respectively. For the direct analysis of the ligand load on the membranes via BCA, the membranes were incubated on a 96-well filter plate with the working reagent and 25 µL of PBS buffer. After incubation, the filter plate was placed on top of a 96-well plate and centrifuged. The resulting permeate was then analyzed with a multiplate reader from the Tecan Group (Männedorf, Switzerland) at 562 nm. The supernatant analysis was carried out via UV-vis at 280 nm.

#### 2.2.6. Dynamic Experiments

The membranes were cut to a diameter of 22 mm to fit into a 25 mm Whatman^®^ plastic filter holder module (Merck, Germany). Subsequently, the membranes were linked to either a syringe which held buffers for adsorption and desorption (1× PBS at pH 7.4 and 50 mM Na-acetate at pH 2.9, respectively) or antibody solutions (i.e., 0.3 mg mL^−1^ of Trastuzumab). These solutions were then carefully passed through manually to establish a semi-dynamic configuration. In this setup, 1 mL fractions from each procedural stage were meticulously collected into distinct tubes. Additionally, an ÄKTA pure system was employed to create a fully dynamic setup. The affinity membranes were applied to the holder module and subjected to flux in respective buffer solutions, including 1× PBS pH 7.4 as equilibration and loading buffer and 50 mM Na-Acetate pH 2.9 as elution buffer. In the syringe setup, each of the functionalized membranes was loaded with 1 mL of Trastuzumab at a concentration of 0.3 mg mL^−1^. After loading, the membranes were washed three times with 1 mL of 1× PBS pH 7.4. The bound antibody was then eluted with 1 mL of 50 mM sodium acetate buffer at pH 2.9, and all fractions were analyzed and balanced via UV-vis. In the dynamic setup, either Trastuzumab or 1:100 diluted human blood plasma was used as the analyte, with respective concentrations. Here, the filter holder was connected to the ÄKTA pure at a volumetric flow rate of 0.5 mL min^−1^. A dead volume of 0.2 mL was determined via 1 M NaCl as tracer solution. The dynamic binding capacity was assessed by directly loading the ligand-immobilized membranes with a 0.3 mg mL^−1^ solution of Trastuzumab until a breakthrough could be observed. The prepared Sartobind^®^ Protein A membranes were in percolation, at a flow rate of 1 mL min^−1^ with a Trastuzumab concentration of 0.3 mg mL^−1^. The membrane was then placed in the same membrane holder module to address similar pressure limitations encountered with our own prepared membranes. Both the dynamic binding capacity (DBC) and load (q*) were calculated by looking at the breakthrough curve. The DBC, at 10% of the obtained max mAU value (DBC10), was calculated via Equation (2).

(2) $${DBC}_{10}=\frac{c_{p}\times Q\times t_{10}}{MV}$$

where Q represents the volumetric flow rate, c_p_ the protein concentration used, t_10_ the time surpassed until 10% breakthrough occurred, and MV the membrane volume previously determined via 1 M NaCl tracer solution. The protein load (q*) was calculated via Equation (3).

(3) $$q^{*}=\frac{c_{p}\times (V_{eq}-V_{0})}{MV}$$

where V_eq_ represents the volume at inflection point and V_0_ the dead volume of the system (measured via NaCl tracer solution). However, the two quantities differ since, for the calculation of q*, the volume at the inflection point (here: idealized assumption with a 50% breakthrough) and the dead volume are used. The DBC10 calculation, on the other hand, only considers the volume after a 10% breakthrough of unbound target protein.

In order to be able to compare two different membranes units, the selectivity factor α is used. It is calculated via the DBC and retention time (RT) of each membrane via Equation (4).

(4) $$\alpha =\frac{{DBC}_{1}}{DBC_{2}}\times \frac{RT_{2}}{RT_{1}}$$

If the α-value is larger than 1, membrane 1 is superior to membrane 2. Moreover, it can bind more of the analyte and elute it faster. If α < 1, membrane 2 is superior to membrane 1. The selectivity factor of α = 1 shows that both membranes have similar performance characteristics.

## 3. Results and Discussion

### 3.1. Qualitative Functionalization Analysis

**Figure 3:**
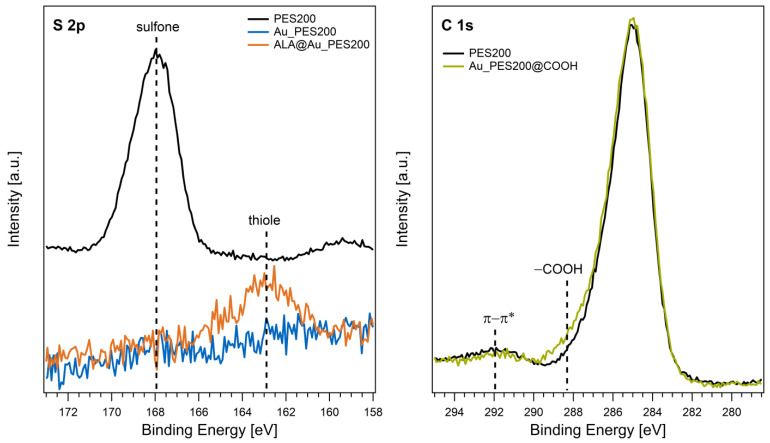
XPS detail spectra of the S 2p (**left**) and C 1s (**right**) core levels acquired from native and Au-sputtered PES200 membranes before and after functionalization.

The left panel of Figure 3 displays the S 2p core level spectra of native PES200, Au_PES200, and ALA@Au_PES200. The signal of the native membrane is centered around 168 eV, which is in good agreement with the binding energies of sulfone groups in PES materials reported in the literature [25]. After the gold-sputtering process, no sulfur species are detected on the surface (Au_PES200). Hence, the S 2p signal of the PES polymer is completely attenuated upon the deposition of an entirely closed Au layer on the membrane surface as observed by surface-sensitive XPS. The functionalization of the material with ALA results in an S 2p signal with a binding energy of 163 eV. This value is characteristic for thiol groups, proving a successful functionalization of the Au surface with sulfur-containing ALA [30].

The functionalization of the PES material with carboxy groups was analyzed using a native PES200 membrane to circumvent the attenuation of PES signals by the Au overlayer. The C 1s core levels before and after e beam treatment are shown in Figure 3 (right panel). Besides the main C 1s signal at 285 eV, a weak and broad peak was found at 292 eV, which is attributed to π-π* shake-up transitions [25]. After functionalization, a shoulder appeared at higher binding energies. A binding energy of ~288.5 eV is typical for carboxy groups in organic matrices [30]. Thus, the additional C 1s component visible after e beam treatment demonstrates the successful introduction of carboxy groups in the PES material.

With the results of the XPS measurements, a successful surface alteration could be observed. With the present carboxy groups, the membranes can be used for Protein A immobilization via NHS/EDC coupling. The ligand load was estimated via a direct BCA analysis of the membranes after protein immobilization (see Figure S7). The Au_PES200@COOH@ProteinA experienced a ligand load of 0.43 g g^−1^, the ALA-functionalized ALA@Au_PES200@ProteinA experienced a ligand load of 0.098 g g^−1^. For the Sartobind^®^ membrane, the BCA assay did not work. BCA as an assay is an error-prone method as it is easily influenced by many factors (e.g., ions in the buffer, surface groups on the material, pH). Since a direct supernatant analysis of immobilized Protein A proved to be challenging, as different reactants are used during the immobilization reactions, only an indirect analysis could be conducted where fluorescence-labelled antibodies, and binding to the ligand could be observed. With a fluorescence scan, the binding of labeled antibody (mAb*) to Protein A-immobilized membranes (i.e., ALA@Au_PES200@ProteinA) could be observed (see Figure S3B). However, the unspecific binding of labeled antibodies to native Au_PES200 is also visible (Figure S3C). This unspecific binding occurs due to the gold–thiol interaction of the present L-cysteine groups in antibodies [31]. The ALA-functionalized membranes did not experience such high unspecific binding; ALA even seemed to act as a preventative measure for antibody–gold adsorption (see Figure S3E).

### 3.2. Evaluation of the Binding and Elution Efficiencies of Ligand-Immobilized Membranes

**Figure 4:**
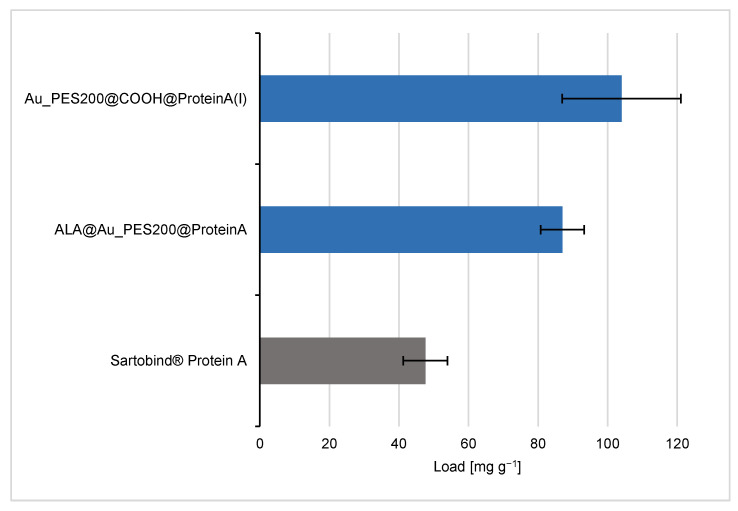
IgG load comparison of differently self-made PES membranes with immobilized engineered Protein A (consisting of 8 polymerized B domains) vs. the Sartobind^®^ Protein A via a static equilibrium binding experiment and supernatant analysis.

The analysis of the binding behavior of the differently functionalized affinity membranes shows different trends. For the sake of comparability, the membranes’ dry weight (see Table S1) is used as reference point to calculate the maximal protein load (see Figure 4). Therefore, the maximum load values are in units of mg of IgG per g of membrane. The Au_PES200@COOH@ProteinA with immobilization protocol (I), where the carboxylated material was used for the immobilization reaction with a longer NHS/EDC protocol, is one of the best candidates with a maximal load q_max_ of 104 mg g^−1^, alongside the ALA-surface-functionalized membranes.

The e beam-carboxylated Au_PES200@COOH membrane, which underwent additional functionalization with ALA (ALA@Au_PES200@COOH), exhibits comparable binding capacities to the solely carboxylated membrane (see Figure S4). This similarity arises from the immobilization process that occurs here primarily due to carboxylation rather than via the ALA functionalization. The results (see Figure S4) indicate that the immobilization reaction (II) exhibits significantly reduced binding capacities compared to method (I). Therefore, its use is not recommended for the immobilization of proteins on membranes. Looking at the blank membranes Au_PES@COOH and Au_PES (see Figure S4), an increase in unspecific antibody binding can be exhibited on the Au_PES@COOH membrane. During the NHS/EDC immobilization reaction, ethanolamine and casein are added to block remaining activated carboxyl groups. Carboxylation increases wettability, therefore changing the solid–liquid interface interaction and thus the accessibility for antibodies to adsorb on the surface [17,21].

These results suggest that the Au_PES200@COOH and ALA@Au_PES200 membranes show higher binding capacities than the commercial Sartobind^®^ Protein A membrane. This difference in binding capacity may be due to the different chemical and physical properties of the membranes, such as their surface area, pore size, and functionalization. However, previous work on this particular version of a Protein A ligand with a polymerized B domain suggested a higher binding capacity for IgG1 due to a better stoichiometry between the ligand and antibody, in comparison to native, recombinant Protein A [14]. The higher binding affinity seen here could therefore be attributed to the different ligands used between the self-made and the commercial membrane. The stochiometric analysis of Protein A/Antibody resulted in 1:11 for the carboxylated membranes and 1:3 for the ALA functionalized membranes. The higher stoichiometry for the carboxylated membranes can be explained by the aforementioned unspecific binding of antibodies to gold (see Figure S3C). Furthermore, some remaining carboxyl groups could act as weak cation exchangers, thus increasing the overall unspecific antibody binding [32]. The ALA functionalization, as a self-assembled monolayer, seems to prevent unspecific binding to gold (see Figure S3E), thus showing a similar stoichiometry as reported in the previous literature [33]. In order to answer whether the higher load was ligand-related or if there were other factors involved leading to the higher binding capacities, a static binding experiment with native, recombinant Protein A was conducted in comparison to the experiments with our engineered Protein A (consisting of 8 polymerized B domains). For the ALA@Au_PES200, which was immobilized with native Protein A, a load of q = 81.0 mg g^−1^ (see Figure S4) could be achieved. These results suggest that it is not the choice of the ligand itself, but rather the herein-demonstrated functionalization process as a whole that leads to higher binding capacities. The functionalization process can involve many factors, such as the chemical and physical properties of the membrane surface, the pore size and distribution, and the chemical functionalization of the membrane.

**Figure 5:**
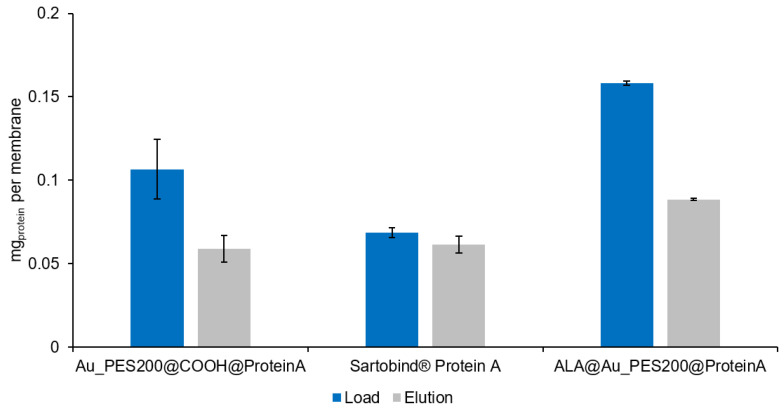
Bound and eluted amount of Trastuzumab in a semi-dynamic syringe setup in triplicates. Each solution manually passed through one single membrane with a diameter of 22 mm for adsorption and elution (1× PBS at pH 7.4 and 50 mM Na-acetate pH 2.9, respectively) at a volume of 1 mL. Initial Trastuzumab concentration of 0.3 mg mL^−1^. Protein concentration measurements by supernatant analysis via UV-vis at 280 nm.

In semi-dynamic experiments (see Section 2.2.6), it was found that the ALA@Au_PES200 membranes, which were functionalized via self-assembled monolayers (SAM) of ALA, exhibited the highest binding efficiencies. This may be attributed to higher mass transfer limitations due to the pores for the e beam-carboxylated Au_PES200@COOH during the dynamic binding process, as the static binding showed that these membranes have the highest binding capacity. In the static setup, mass transfer limitations are not of concern; thus, the available binding sites within the membrane’s pores can be accessed. Notably, only the gold surface of the ALA@Au_PES200 membrane was functionalized (see Figure 1A), which could explain its higher binding capacity in the dynamic setup, due to its accessibility and reduced mass transfer limitations. Nevertheless, in the dynamic setup, the Au_PES200@COOH membranes still showed a better binding capacity than the Sartobind^®^ membranes (see Figure 5). With an increased residence time, the dynamic binding capacity of Au_PES200@COOH could be improved, as was seen in a previous study by Boi et al. [7]. Regarding elution, the industry-standard Sartobind^®^ A showed the best elution (grey graph in Figure 5), since almost 90% of bound antibodies could be eluted. However, the Au_PES200@COOH membrane showed a competitive 50–70% elution. The lower elution efficiency can be attributed to the gold layer present in the PES membranes. Gold is prone to the unspecific binding of antibodies, reducing the overall elution efficiency [34]. Furthermore, a low amount of unreacted carboxylic groups has to be considered, creating an ion exchange effect, which could directly impact how efficiently the elution process works. However, with the initial higher binding capacity, the better elution efficiency of the commercial membrane is amortized. Furthermore, the effect of unspecific binding is reduced with increased cycles, as the unspecific binding sites will eventually be occupied.

About 60 µg of antibodies can be eluted from the Au_PES200@COOH@ProteinA membrane and the Sartobind^®^, whereas 90 µg could be eluted from the ALA@Au_PES200@ProteinA membrane. The difference between the two self-made membranes can be explained by looking at the static experiments (see Figure 4). Because ALA-functionalized membranes have ligands positioned on the surface, antibodies can readily bind to them without encountering mass transfer limitations. In contrast, the Au_PES200@COOH@ProteinA membrane carries the ligand deeper within the material, resulting in a greater mass transfer limitation effect. This can be reduced by increasing the residence time on the membranes. Nevertheless, both membranes exhibited a higher antibody-binding capacity compared to Sartobind^®^, which is consistent with the findings from the static experiments. The elution efficiency for both self-made membranes, even without optimizing residence time, was around ~55%, making them a promising foundation for initiating a practical dynamic setup.

### 3.3. Dynamic Processing of New Membranes with Purified Trastuzumab

**Figure 6:**
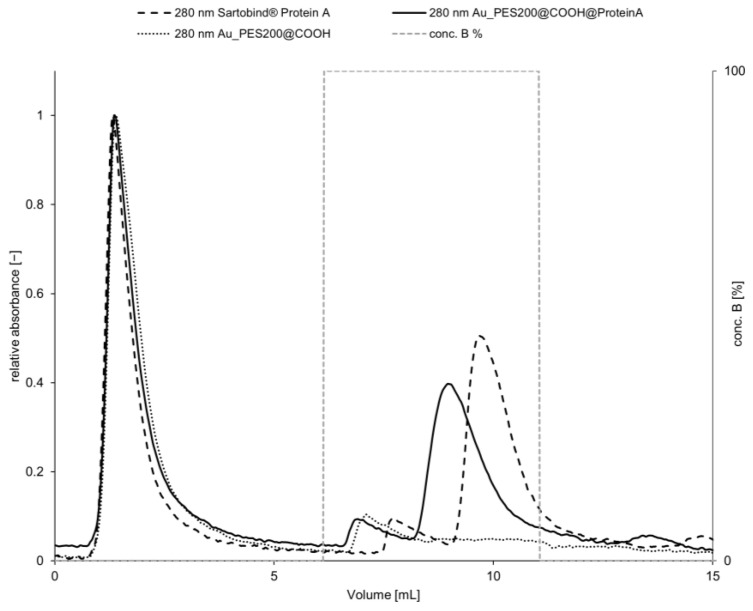
Chromatogram of the capture in 1× PBS at pH 7.4 and release in 50 mM Na-acetate at pH 2.9 of 0.264 mg mL^−1^ Trastuzumab on Au_PES200@COOH@ProteinA and Sartobind^®^ Protein A with a Au_PES200@COOH membrane without a ligand as a blank. One single membrane with a diameter of 22 mm, flow rate = 1 mL min^−1^, and RT of 3.55 min for the Sartobind^®^ membrane and RT of 2.85 min for the Au_PES200@COOH@ProteinA.

After proving the binding and eluting behavior, experiments were transferred in a dynamic setup under process conditions. For this experiment, a Trastuzumab concentration of 0.264 mg mL^−1^ was used. After NaCl tracer analysis, the membrane module volume (MV) was calculated to be 0.3 mL. Looking at the chromatogram, a clear capture and release is evident and proves the applicability of the designed membrane in a dynamic process (see Figure 6). The control membrane Au_PES200@COOH, without any ligand protein, shows no elution peak at 280 nm after antibody contact; therefore, the Trastuzumab was not captured. In traditional column chromatography, the dynamic binding capacity DBC10 indicates the amount of protein that can be purified from a particular resin volume and serves as a process parameter allowing for comparison of different materials and processes. Breakthrough analysis is the most popular method for measuring it [35]. Commercially available bed-based chromatography products with Protein A, such as MabSelect™ by Cytiva, have dynamic binding capacities of 35–65 mg mL^−1^ and retention times (RT) of 2.4–6 min; Monofinity A Resin™ by Genscript achieved a DBC10 of 30 mg mL^−1^ with a RT of 4 min or even monoliths such as CIMac™ by BIA Separations of >5 mg mL^−1^ [6]. In a recent study by Osuofa and Husson [36], different new affinity membrane materials were tested regarding performance. The four tested membranes comprised a composite membrane by Gore, an electrospun fiber by Cytiva, an affinity membrane from Purilogics which is based on a convection-based flow, and the Sartobind^®^ Protein A membrane. Among all listed membranes, the only membrane material that is comparable with the technology used in this approach is the Sartobind^®^ membrane. The other mentioned membranes have structurally changed properties, leading to higher binding capacities, and probably exhibit a certain degree of higher mass transfer limitations. Membranes and monolithic materials will always have a lower binding capacities than bead-based chromatography, as their specific surface area is significantly lower [4]. However, in the overall yield, this is amortized due to a lack of mass transfer limitations for membranes [3].

For membrane chromatography, similar process characteristics should be applied; although a decrease of mass transfer limitations is present, the overall idea of the DBC as a process parameter is still a valid process characteristic measurement [37]. At a flow rate of 5 to 10 mL min^−1^ and a membrane volume of 2 mL (20 stacked membranes), a DBC10 of 5–7.5 mg polyclonal antibodies per mL membrane is reached using the calculated values from the Sartorius product brochure [38]. During this study, the DBC10 of a commercial membrane (Sartobind^®^ Protein A) where only one single sheet was cut to a diameter of 22 mm was compared to a self-made membrane (Au_PES200@COOH@ProteinA). We can see that the commercial membrane has a higher dynamic binding capacity of 2.465 ± 0.247 mg protein per mL at a retention time (RT) of 3.55 min, in comparison to the self-made membrane of with a binding capacity of 1.320 ± 0.148 mg protein per mL at a RT of 2.85 min and a max load (q*) of 2.929 ± 0.297 mg protein per mL and 1.576 ± 0.218 mg protein per mL, respectively. The rarely reduced dynamic binding capacity of the in-house prepared membrane has to be attributed to the larger pore diameter of the Sartobind^®^ Protein A membrane and the lower mass transfer limitations. The selectivity factor α = 0.67 for both membranes shows that the commercial membrane offers better binding and elution in a dynamic process; however, with increased retention time, the in-house membrane would offer the same efficiency. Despite having a lower dynamic binding capacity, it should be noted that the value of the in-house-prepared membrane remains comparable to that of the commercial membrane. These findings are consistent with the trends observed during the static binding experiments.

### 3.4. Stability of the Functionalized Membranes (Reusability and Storage)

**Figure 7:**
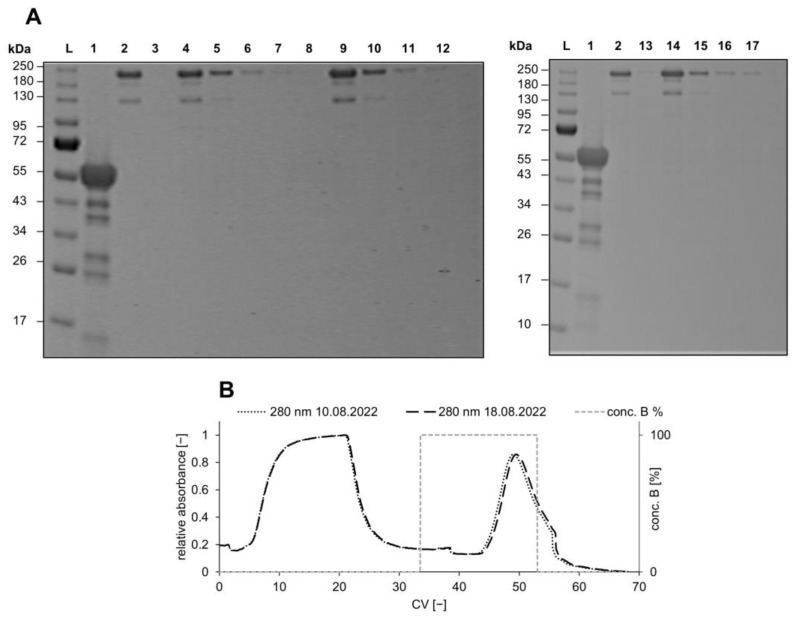
(**A**) Three consecutive runs with Au_PES200@COOH@ProteinA membrane analyzed by non-reducing 12% SDS-PAGE; samples were prepared with non-reducing loading dye and incubated at 80 °C for 5 min. (L) colored pre-stained ladder (mAb) Trastuzumab; (W) wash—1× PBS pH 7.4; (L1–3) load one to three—0.264 mg mL^−1^ Trastuzmab; (W1–3) wash after sample application—1× PBS pH 7.4; (E1–3) elution—50 mM Na-acetate pH 2.9; (**B**) chromatogram of a dynamic performance test of two differently aged Au_PES200@COOH@ProteinA membranes at 1 mL min^−1^, loaded with 3 mL of 0.264 mg mL^−1^ Trastuzmab.

It is essential to test the reusability and storage stability of protein-immobilized membranes over time, particularly for their real-life applications and process cost estimations. If the membrane loses its activity or deteriorates over time, it not only reduces the product yield but also increases the processing cost, leading to financial losses. Additionally, the functionalized membrane’s storage conditions, such as temperature and humidity, can affect its stability, making it crucial to assess its long-term stability. Previous studies showed the influence of storage temperature on proteins’ stability. Overall, they concluded that storage temperatures of −20 to 4 °C and humidities of below 30% showed the best results for immobilized enzymes [39,40]. Here, the membranes were stored in PBS buffer solution for 7 days after ligand immobilization at 4 °C. The results of these storage conditions on the performance of the process can be seen in the chromatogram in Figure 7B.

The developed Au_PES200@COOH@ProteinA membranes seem to be reusable without detectable performance reduction over several cycles and after seven storage days. The fresh membrane was loaded and eluted consecutively three times with mABs. The loading and elution were then repeated after seven storing days. The fresh membrane showed identical behavior during all three cycles of equilibrate, load, wash, and elute and was analyzed via qualitative SDS-PAGE (see Figure 7A). Due to the heating of the SDS-PAGE samples, monoclonal antibodies tend to partially denature, resulting in heterogeneities [41]. Great aging performance of the membrane over an extended storing period in 1× PBS at pH 7.4 for 7 days was shown in comparison to a “fresh” membrane in overlaid chromatogram analysis (see Figure 7B). The older membrane performed just as well as the “fresh” membrane, suggesting that the immobilized Protein A stays “active” even after prolonged storage. These results provide a preliminary idea on the membranes’ performance. However, in order for them to be used in a real industrial application, a higher cycle number and storage time have to be assessed.

### 3.5. Application to a Real Process: Antibody Purification from Fresh Frozen Human Blood Plasma

After establishing the proof of concept of the newly developed membrane, it is crucial to test its performance in a complex medium. This testing provides a first glimpse into a real-life application and offers new insights into the membrane’s behavior in more realistic conditions. The behavior of the immobilized protein on the membrane can vary significantly in complex media, such as blood plasma or cell culture media, compared to simpler solutions [42]. Therefore, testing in complex media provides a more accurate representation of the membrane’s behavior in real-life scenarios, such as medical diagnostics, biopharmaceutical production, and environmental monitoring. Similar studies, such as the one by Schwark et al., looked at epitope-imprinted membranes, where a purity of 88% could be achieved [43]. Barroso et al. was able to construct an affinity membrane with a synthetic ligand where ~33% of the antibodies remained on the membrane after elution [44]. Here, the process was carried out in comparison to the Trastuzumab experiments on a Au_PES200@COOH@ProteinA membrane and a Sartobind^®^ Protein A membrane (see Figure S6). However, as an analyte, a 1:100 dilution of human blood plasma was used.

**Figure 8:**
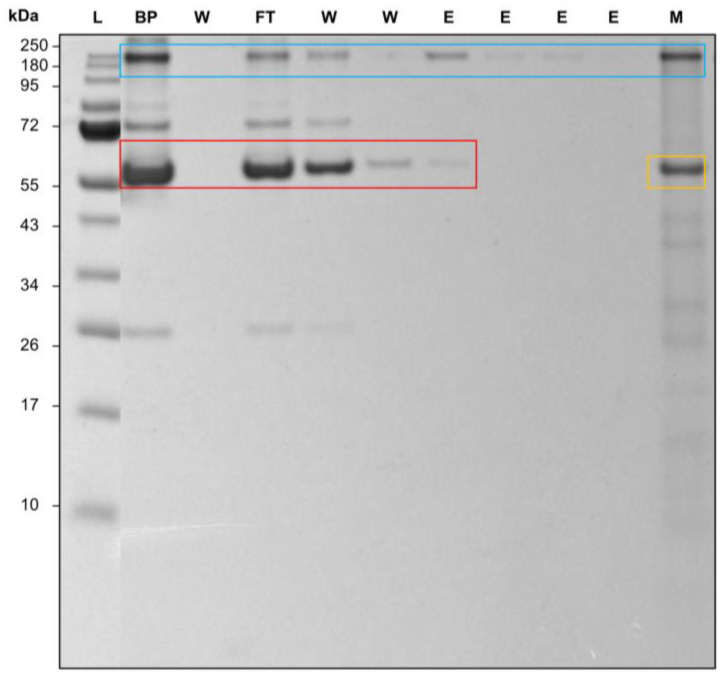
Image of 12% SDS-PAGE of an antibody’s purification process from human blood plasma. The red box shows serumalbumin, the blue box antibodies, and the yellow box the Protein A ligand on the membrane. (BP) 1:100 diluted blood plasma; (W) wash with 1× PBS pH 7.4; (FT) flowthrough during the loading step; (E) elution with 50 mM Na-acetate pH 2.9; (M) used membrane after the process, submerged and cooked at 95 °C for 5 min in loading buffer.

A purification of antibodies was possible with both membranes, even with a complex protein mixture such as human blood plasma. For the Au_PES@COOH@ProteinA membrane, antibodies can be found at 250 kDa (Figure 8 blue box) and at 66 kDa for serumalbumin (Figure 8 red box) [45]. The increase in size of the antibodies is again due to the partial unfolding of the protein during the heating process during sample application [41]. The second elution fraction (the elution peak maximum) showed no remaining plasma proteins; therefore, a purity of over 95% via Imagequant densitometric analysis was achieved. At the beginning of the elution, lane 7 showed some coeluting serumalbumin, resulting in a purity of 89% via Imagequant analysis. A gradient elution would be able to remove the unspecific bound albumin. This also occurred in the elution fraction on the Au_PES200 membranes (see Figure S5) and has to be attributed to the protein interacting with the material. Albumin has been shown to interact with gold [46]; therefore, additionally to the blocking of the membrane, the co-functionalization with ALA could hinder the protein–gold interaction as well. The analysis of the membranes post-process via SDS in lane 11 showed the remaining antibodies, and this is consistent with the results of the semi-dynamic syringe setup (see Figure 5) and the ligand-immobilized Protein A on the membrane (yellow box Figure 8). An improvement of the elution conditions is therefore advisable. Overall, testing in complex media confirmed the proof of concept and offers additional validation to this work. A leaching of the immobilized Protein A ligand is not visible in the SDS-PAGE. However, at very low concentrations of the leached ligand, no band could be visible.

## 4. Conclusions

In conclusion, during this study, we could successfully detect the carboxylation of the membranes and demonstrate the immobilization of the affinity ligand, Protein A. Moreover, we established a proof of concept for using these membranes in an affinity membrane chromatographic process. During this study, the gold-sputtered polyethersulfone membrane with a pore diameter of 200 µm was used as the stationary phase. The Protein A ligand was immobilized on the membranes and tested in a variety of setups. Both self-made affinity membranes (ALA@Au_PES200@ProteinA and Au_PES200@COOH@ProteinA) showed promising capabilities as stationary phases. The selected affinity membrane Au_PES200@COOH@ProteinA, with the immobilized Protein A variant consisting of polymerized B domains, displayed the highest static binding capacity of 104 mg g^−1^, therefore rivaling the commercially available Sartobind^®^ Protein A affinity membrane from Sartorius. The immobilization of native Protein A still showed a higher binding capacity of q = 81.0 mg g^−1^ on ALA@Au_PES200, therefore attributing the load increase to an overall more efficient immobilization reaction and not the choice of ligand itself. With the selected best candidate, the capture and release of monoclonal antibodies in both static and dynamic experiments could be demonstrated, and a stable membrane chromatographic process for the purification of antibodies could be established. We were able to purify antibodies from human blood serum, resulting in product purities of >95%.

Immobilizing proteins on membranes for a membrane chromatography process can offer several advantages over traditional column chromatography, including faster mass transfer rates, reduced sample dilution, reduced column fouling, and easy scalability. In particular, when looking at a whole process, buffer consumption, timing, and life cycles play an important role in relation to the overall production costs. In addition to the advantages of membrane chromatography over traditional column chromatography, the use of sputtered gold in the Au_PES200@COOH membrane offers new opportunities. This layer makes it a potential candidate for electrosorptive processes, as the gold layer can serve as a conductive material. This opens up exciting possibilities for utilizing Au_PES200@COOH@ProteinA and ALA@Au_PES200@ProteinA in a variety of controlled assays and other applications.

## Data Availability

Data will be made available upon reasonable request. The data are not publicly available due to ongoing researches using a part of data.

## Supplementary Materials

The following supporting information can be downloaded at: https://www.mdpi.com/article/10.3390/membranes14020031/s1, Figure S1. TSA of (A) B8(RH)_4_ and (B) recombinant wildtype Protein A in different buffers. Figure S2. XPS spectra of different membranes. Figure S3. Fluorescence imaging via Amersham Typhoon scanner, CY2 emission filter and Imagequant software of (A) blank Au_PES200 membrane, (B) ALA@Au_PES200@ProteinA@Trastuzumab*, (C) unfunctionalized Au_PES200@Trastuzumab*, (D) Au_PES200@COOH@Trastuzumab*, (E) ALA@Au_PES200@Trastuzumab*, (F) Au_PES200@COOH@ProteinA@Trastuzumab*, and (G) Au_PES200@COOH. Figure S4. IgG load comparison on different membrane candidates in blue with our engineered Protein A (consisting of 8 polymerized B domains) and in red with native Protein A; blanks do not contain any ligand proteins. Figure S5. 12% SDS-PAGE of a process with Au_PES200@COOH@ProteinA and Au_PES200. (Load) with 1:100 diluted human blood serum; (W) wash with 1× PBS pH 7.4 three times after loading and one time after the elution; (E) elution with 50 mM Na-acetate pH 2.9. Figure S6. Image of 12% SDS-PAGE of a process with a Sartobind^®^ Protein A membrane. (FT) Flowthrough of 1:100 diluted human blood serum; (W) wash with 1× PBS pH 7.4 three times after loading; (E) elution with 50 mM Na-acetate pH 2.9. Figure S7. Direct BCA of the Protein A load after ligand immobilization. Table S1. Weight measurements of dry membrane.
