# Supplementary material for: Development of a New Affinity Gold Polymer Membrane with Immobilized Protein A
Source: Membranes (Basel). 2024 Jan 24;14(2):31. doi: 10.3390/membranes14020031 (PMC10890041; doi:10.3390/membranes14020031)
Supplement: Supplementary file 1 [file membranes-14-00031-s001.zip › membranes-2758982-supplementary.pdf]

Supplementary information:

Figure S1: Thermal shift assay to assess protein stability

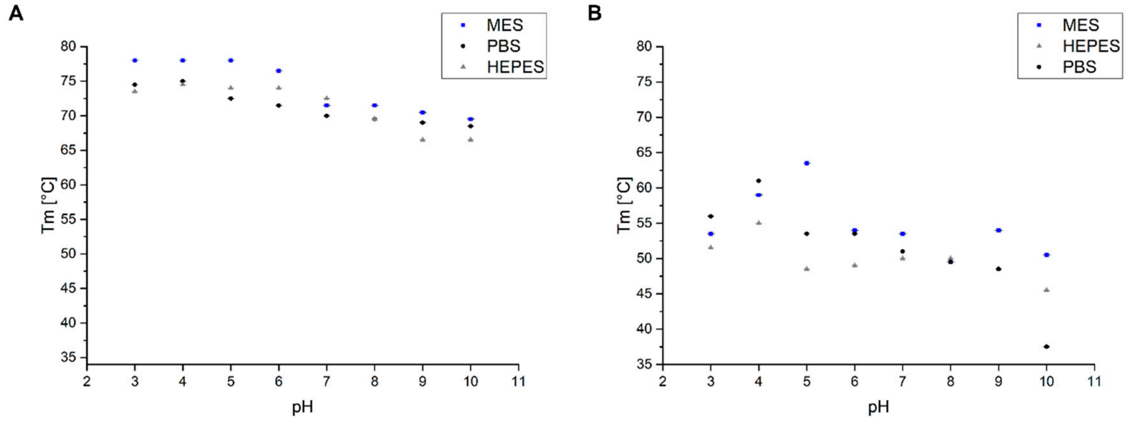

Figure S1 TSA of A) B8(RH)$_4$ and B) recombinant wildtype Protein A in different buffers

A thermal shift assay comparing the in this study used Protein A ligand B8(RH)$_4$ and native Protein A. The polymerized Protein A ligand showed a higher stability in all buffer systems.

Table S1: Weight measurements of membranes

Table S1 weight measurements of dry membranes

**Membrane**	**Weight d = 6 mm [mg]**
Au_PES200@COOH	0.533 ± 0.057
PES200@COOH	0.9 ± 0.1
Au_PES200	0.767 ± 0.115
Sartobind® Protein A	1.78 ± 0.179

Figure S2: XPS qualitative analysis of functionalization success

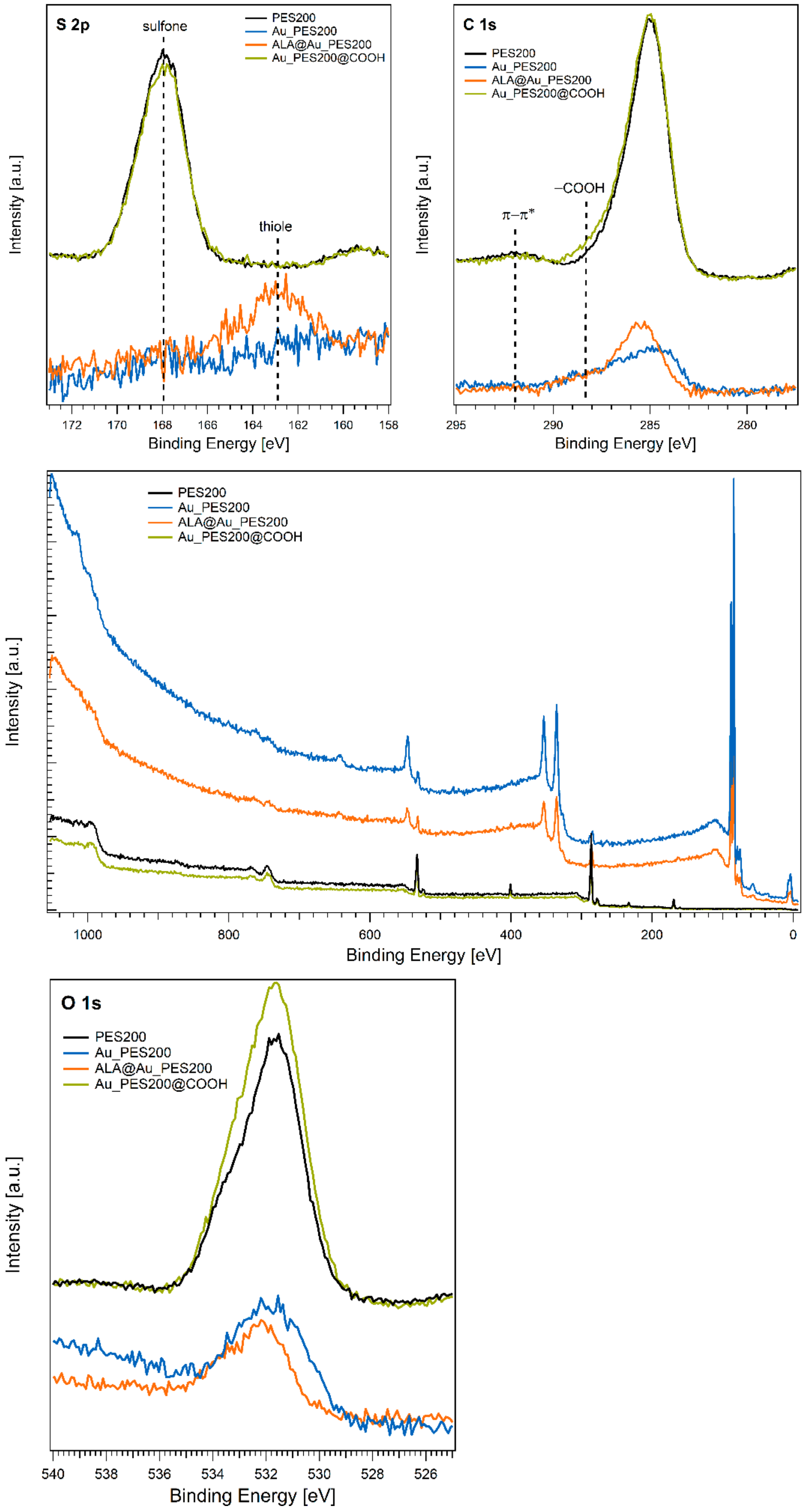

Figure S2 XPS spectra of different membranes

Figure S3: Qualitative analysis of successful protein immobilization via fluorescence scan of Trastuzumab*

To visualize successful protein immobilization a fluorescence scan experiment was conducted for the ALA functionalized membranes. The unfunctionalized Au_PES200 membrane acts as a blank, since it does not show any fluorescence, which is expected as no labeled antibody was added. The blank sample without any fluorescence was subtracted from both sample membranes. The Protein A functionalized ALA@Au_PES200@ProteinA@IgG* shows a strong fluorescence with a median intensity of 7910.31 AU, indicating the presence of a sufficient quantity of labelled antibody. The control without immobilized Protein A, Au_PES200@IgG* shows some fluorescence of 2615.88 AU (Figure S3 C), the amount is lower than the ligand immobilized sample, yet still indicating some unspecific binding events of the antibody.

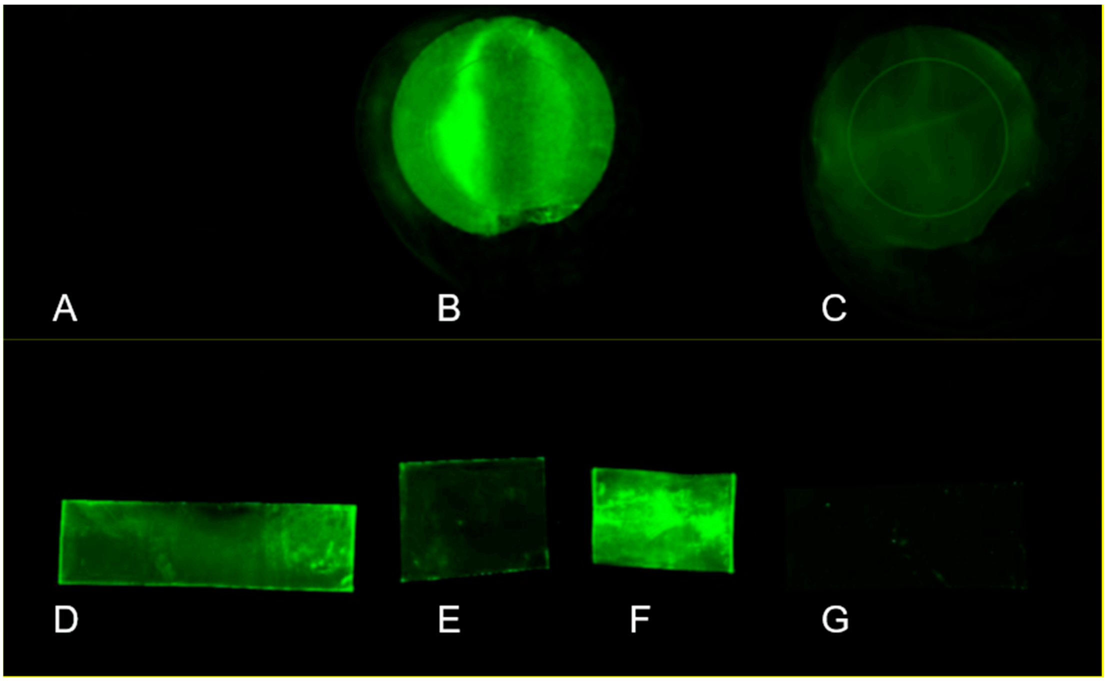

Figure S3 Fluorescence imaging via Amersham Typhoon scanner, CY2 emission filter and Imagequant software of A) blank Au_PES200 membrane B) ALA@Au_PES200@ProteinA@Trastuzumab C) unfunctionalized Au_PES200@Trastuzumab* D) Au_PES200@COOH@Trastuzumab* E) ALA@Au_PES200@Trastuzumab* F) Au_PES200@COOH@ProteinA@Trastuzumab* G) Au_PES200@COOH*

By directly comparing two samples using fluorescence scanning, it was found that the antibody capture increased by a factor of 3:1 due to the immobilized Protein A ligand. This increase indicates the presence of the ligand protein and serves as first evidence of successful protein immobilization.

In Figure S3 D, Au_PES200@COOH@Trastuzumab* displays an average intensity of 2441.50 AU. Figure S3 E illustrates the ALA@Au_PES200@Trastuzumab* with an average intensity of 670.99 AU, while in Figure S3 F, the ALA@AU_PES200@ProteinA@Trastuzumab* exhibits an average intensity of 6138.61 AU. Additionally, Figure S3 G portrays the untreated membrane with an average intensity of 367 AU.

Figure S4: SBC of Trastuzumab on different membrane candidates

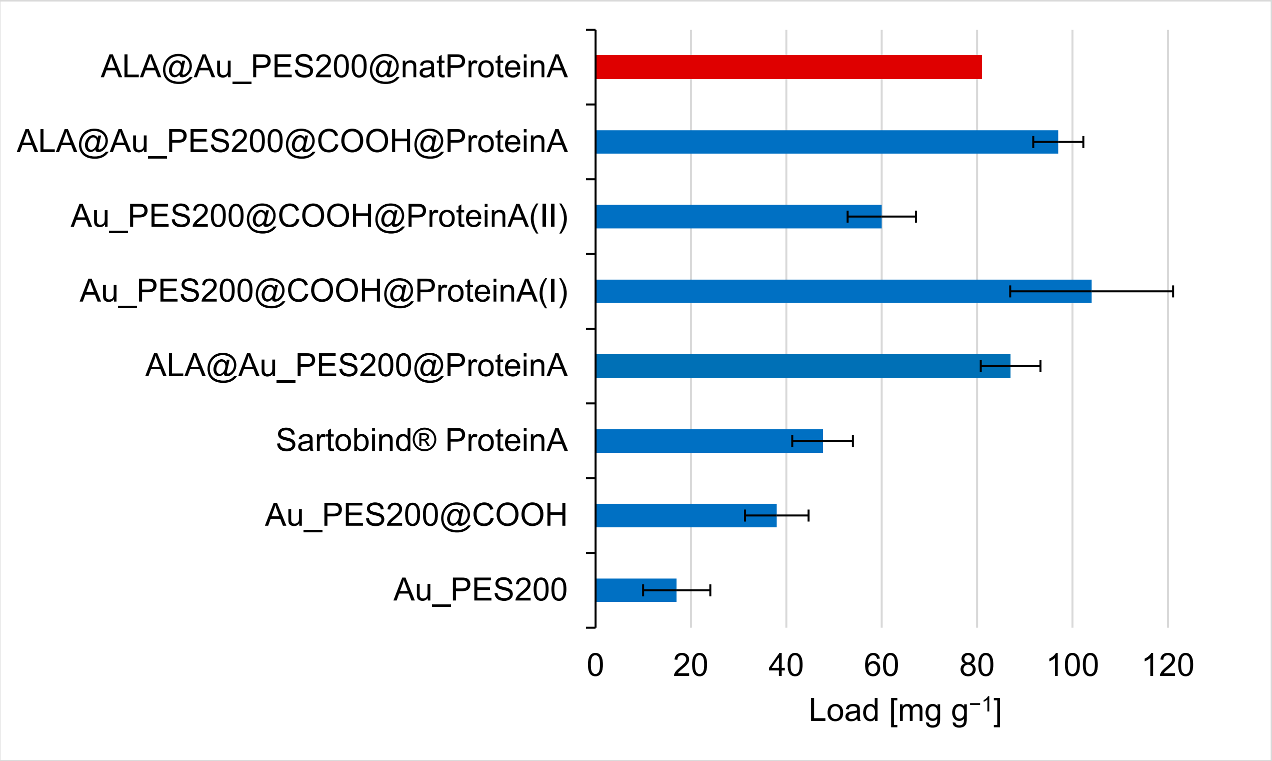

Figure S4 IgG load comparison on different membrane candidates in blue with our engineered Protein A (consisting of 8 polymerized B domains) and in red with native Protein A, blanks do not contain any ligand proteins

During static binding capacity tests with the wildtype native recombinant Protein A from *S. aureus* (natProteinA), was used to see whether the increase in binding capacity had something to do with the eight-fold polymerization of the B-domain in the polymerized Protein A ligand that was used in the experiments during this study. Furthermore, the hypothesized hydrophillization of the carboxylated Au_PES200@COOH is apparent when compared to Au_PES200. The influence of the two functionalization strategies is apparent, when comparing both strategies with each other (I, II).

Figure S5: SDS-PAGE of Protein A immobilized and no ligand immobilized Au_PES200 membranes

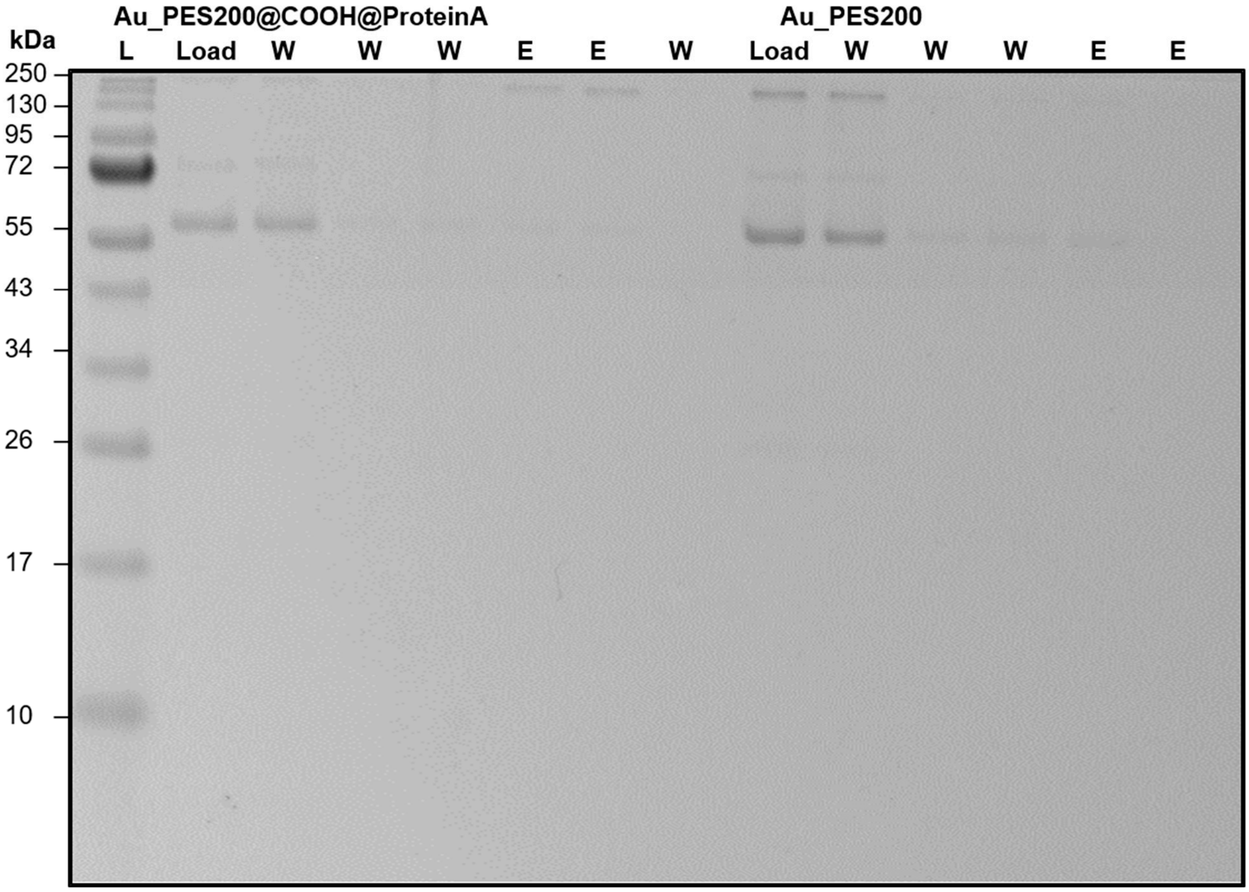

Figure S5 12% SDS-PAGE of a process with Au_PES200@COOH@ProteinA and Au_PES200. Load) with 1:100 diluted human blood serum, W) wash with 1×PBS pH 7.4 – three times after loading and one time after the elution, E) elution with 50 mM Na-acetate pH 2.9

The membrane with immobilized Protein A (Au_PES200@COOH@ProteinA) shows eluted antibodies during the elution steps. In contrast, the Au_PES200 without immobilized ligand shows no antibody bands.

Figure S6: SDS-PAGE of a single 22 mm diameter Sartobind® Protein A membrane in a process with human blood serum

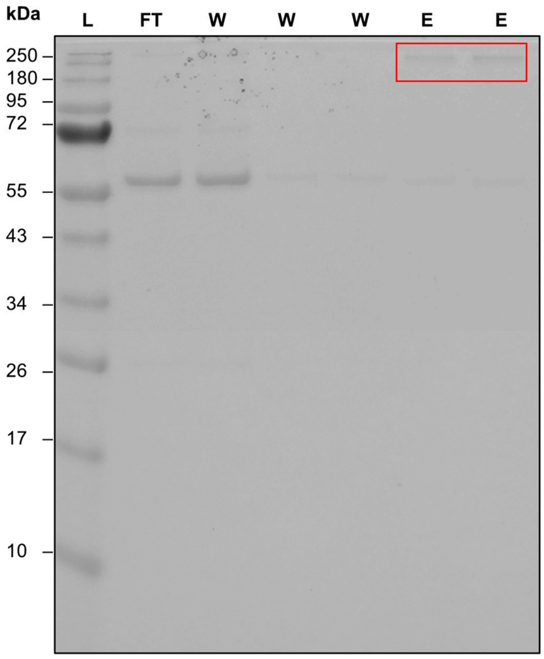

Figure S6 12% SDS-PAGE of a process with a Sartobind® Protein A membrane. FT) Flowthrough of 1:100 diluted human blood serum, W) wash with 1×PBS pH 7.4 three times after loading, E) Elution with 50 mM Na-acetate pH 2.9

Figure S7 direct BCA analysis of membranes right after Protein A immobilization

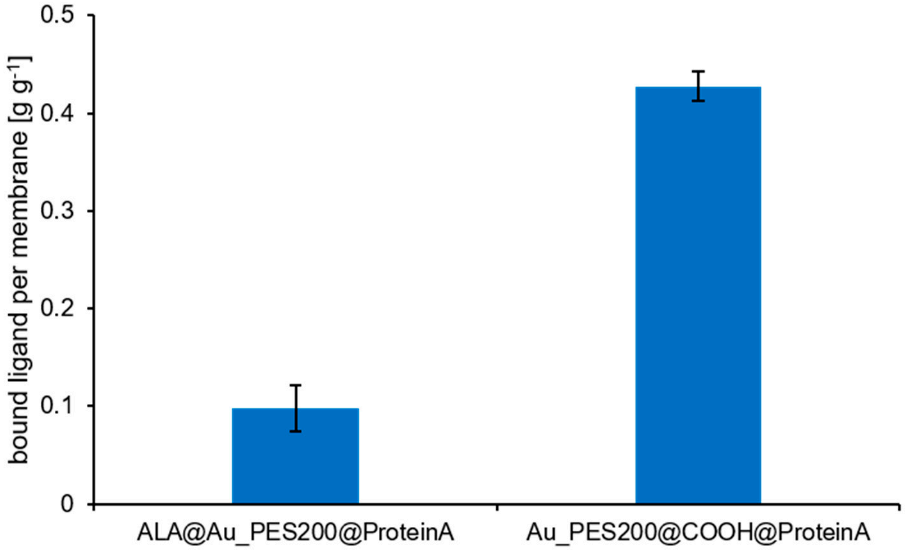

Figure S7 direct BCA of the Protein A load, after ligand immobilization

Direct BCA resulted in a measured amount of Protein A on the ALA functionalized membrane of 0.098 g g^{-1} and for the carboxylated membrane of 0.43 g g^{-1}. For the stochiometric analysis the molar mass of the Protein A ligand (8-fold polymerized B domain of Protein A) and IgG were calculated. For the Protein A ligand a size of 57 kDa was calculated via aminoacid sequence analysis via ProtParam by Expasy. For IgG a size of 150 kDa was used for the calculation.
